# Generation of an induced pluripotent stem cell line (TRNDi008-A) from a Hunter syndrome patient carrying a hemizygous 208insC mutation in the *IDS* gene

**DOI:** 10.1016/j.scr.2019.101451

**Published:** 2019-04-25

**Authors:** Junjie Hong, Miao Xu, Rong Li, Yu-Shan Cheng, Jennifer Kouznetsova, Jeanette Beers, Chengyu Liu, Jizhong Zou, Wei Zheng

**Affiliations:** a National Center for Advancing Translational Sciences, National Institutes of Health, Bethesda, MD, USA; b iPSC core, National Heart, Lung and Blood Institute, National Institutes of Health, Bethesda, MD, USA; c Transgenic Core, National Heart, Lung and Blood Institute, National Institutes of Health, Bethesda, MD, USA

## Abstract

Mucopolysaccharidosis Type II (MRS II), also known as Hunter syndrome, is a rare X-linked genetic disease caused by mutations in the *IDS* gene encoding iduronate 2-sulfatase (I2S). This is a multisystem disorder with significant variation in symptoms. Here, we document a human induced pluripotent stem cell (iPSC) line generated from dermal fibroblasts of a patient with Hunter syndrome containing a hemizygous mutation of a 1 bp insertion at nucleotide 208 in exon 2 of the *IDS* gene. The generation of this line will allow development of cell-based models for drug development, as well as the study of disease pathophysiology.

**Table:** Resource table.

Unique stem cell line identifier	TRNDi008-A
Alternative name(s) of stem cell line	HT525A
Institution	National Institutes of Health National Center for Advancing Translational Sciences Bethesda, Maryland, USA
Contact information of distributor	Dr. Wei Zheng Wei.Zheng@nih.gov
Type of cell line	iPSC
Origin	Human
Additional origin info	Age: 3-year-old Sex: Male Ethnicity: Caucasian
Cell Source	Skin fibroblasts
Clonality	Clonal
Method of reprogramming	Integration-free Sendai viral vectors
Genetic Modification	NO
Type of Modification	N/A
Associated disease	Mucopolysaccharidosis Type II
Gene/locus	Gene: IDS Locus: Xq28 Mutation: c.208insC (p. H70PfsX29)
Method of modification	N/A
Name of transgene or resistance	N/A
Inducible/constitutive system	N/A
Date archived/stock date	2018
Cell line repository/bank	N/A
Ethical approval	NIGMS Informed Consent Form was obtained from patient at time of sample submission. Confidentiality Certificate: CC-GM-15-004

## Resource utility

The human induced pluripotent stem cells (hiPSC) described here is a useful tool that can be used to investigate disease phenotype and pathophysiology. As a potential cell-based disease model, these cells can be employed for drug development for the treatment of patients with MPS II.

## Resource details

MPS II is a rare X-linked genetic disease caused by mutations in the *IDS* gene encoding iduronate 2-sulfatase (I2S). The I2S enzyme is involved in the lysosomal degradation of two kinds of glycosaminoglycans (GAGs): heparan sulfate and dermatan sulfate (Hopwood et al., 1993). Malfunction of I2S leads to progressive accumulation of GAGs in tissues and organs, causing a variety of clinical symptoms in the patients. The typical manifestations of this disease include respiratory obstruction, cardiomyopathies, joint stiffness and hepatosplenomegaly. Some patients also have central nervous system (CNS) involvement, such as progressive neurological decline and cognitive impairment (Wraith et al., 2008; Whiteman and Kimura, 2017).

**Fig. 1.**
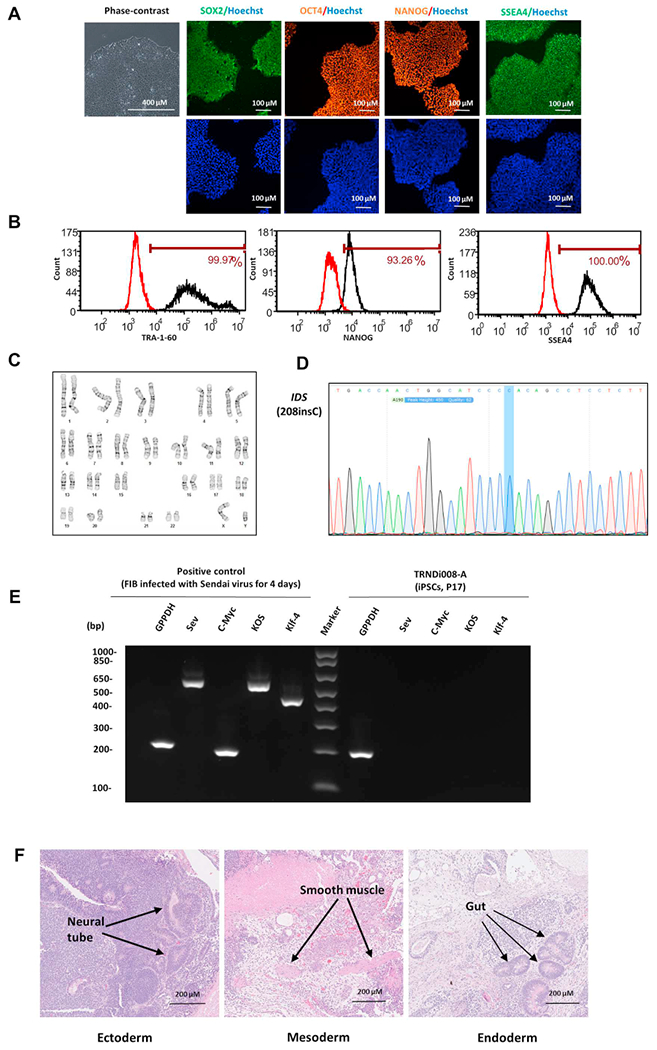
Characterization of TRNDi008-A iPSC line A) From left to right: phase contrast imaging of TRNDi008-A colonies; immunostained TRNDi008-A iPSCs expressing SOX2, OCT4, NANOG and SSEA4. Hoeschst (blue) was used to label the nucleus. B) Pluripotency protein markers (TRA-1-60, NANOG and SSEA4) were assessed by flow cytometry. C) G-banding karyotype analysis confirmed normal karyotype (46, XY). D) Sanger sequencing was used to confirm the mutation in exon 2 of the IDS gene (208insC). E) RT-PCR verification of Sendai virus clearance in TRNDi008-A iPSC line. SeV transduced fibroblasts were used as positive control. F) Histological characterization of teratoma formation, showing normal ectoderm, endoderm and mesoderm differentiation.

In this study, a human iPSC line was established from the fibroblasts of a 3-year-old male patient (GM13203, Coriell Institute) carrying a hemizygous mutation of a 1 bp insertion at nucleotide 208 in exon 2 (208insC) of the *IDS* gene, resulting in a frameshift with a premature stop codon (H70PfsX29). As described previously (Beers et al., 2015), the OCT3/4, KLF4, SOX2 and C-MYC pluripotency transcription factors were employed to transduce the patient fibroblasts into an iPSC line, named TRNDi008-A. The iPS cells exhibited classical embryonic stem cell morphology (Fig. 1A), characterized by immunofluorescence staining and flow cytometry analysis and expressed major pluripotent protein markers of NANOG, SOX2, OCT4, SSEA4 and TRA-1-60 (Fig. 1A, B). Furthermore, G-banded karyotyping at passage 11 (Fig. 1C) confirmed a normal karyotype (46, XY). The mutation (208insC) in the *IDS* gene was also verified by Sanger sequencing of the PCR product harboring the single nucleotide variation (SNV) (Fig. 1D). Sendai virus vector (SeV) clearance was determined with reverse transcription polymerase chain reaction (RT-PCR) using SeV-specific primers and the vector disappeared by passage 15 (Fig. 1E). Mycoplasma status was confirmed to be negative (Supplementary Fig. S1) and the cell line was authenticated using STR DNA profiling analysis, which demonstrated matching genotypes at all 18 loci examined (information available with the authors). Finally, pluripotency of TRNDi008-A was confirmed by a teratoma formation experiment, which exhibited the ability to differentiate into all three germ layers (ectoderm, neural tube; mesoderm, smooth muscle; endoderm, gut) *in vivo* (Fig. 1F)(Table 1).

## Materials and methods

### Cell culture

Patient-derived fibroblasts (GM13203, Coriell Institute) were cultured in DMEM supplemented with 10% fetal bovine serum, 100 units/ml penicillin and 100 μg/ml streptomycin in a humidified incubator with 5% CO_2_ at 37 °C. Human iPS cells were cultured in StemFlex medium (ThermoFisher) on matrigel (Corning, 354,277)-coated plates at 37 °C in humidified air with 5% CO_2_ and 5% O_2_. Cells were passaged with 0.5 mM Ethylenediaminetetraacetic acid (EDTA) upon approaching 80% confluency.

### Reprogramming of human skin fibroblasts

As described previously (Li et al., 2019), patient fibroblasts were reprogrammed into iPS cells using non-integrating Sendai virus vector technology (A16517, ThermoFisher).

### Genome analysis

Genomic analysis of IDS variants was performed by Applied StemCell (Milpitas, California). Briefly, QuickExtract^™^ DNA Extraction Solution (Lucigen) was used to extract genomic DNA from TRNDi008-A cells. PCR amplifications (MyTaq^™^ Red Mix, Bioline) were carried out on a T00 Thermal Cycler (Bio-Rad) using the following program: 95 °C, 2 mins; 35 cycles of [95 °C, 15 s; 60 °C, 15 s; 72 °C], elongation duration varies by amplicon size, 72 °C 5 mins; 4 °C, indefinite. Sanger sequencing analysis was performed for genotyping of the hemizygous mutation of a 1 bp insertion at nucleotide 208 in exon 2 of the *IDS* gene. The specific primers for gene amplification and sequencing are listed in Table 2.

### Immunocytochemistry

Patient-derived iPSCs were fixed for 15 mins in 4% paraformaldehyde, washed with Dulbecco’s Phosphate-Buffered Saline (DPBS), permeabilized with 0.5% Triton X-100 in DPBS (10 mins), followed by incubation with Image-iT^™^ FX signal enhancer (ThermoFisher) for 40 mins at ambient temperature in a humidified environment. Primary antibodies (SOX2, OCT4, NANOG and SSEA4) were diluted in the Image-iT^™^ FX signal enhancer blocking buffer and then incubated with the cells overnight at 4 °C. Following a DPBS wash, corresponding secondary antibodies conjugated with Alexa Fluor 488 or Alex Fluor 594 were added and incubated for 1 h at ambient temperature (Table 2). Cells were stained with Hoechst 33342 (15 mins), washed, and imaged using an INCell Analyzer 2200 (GE Healthcare) and a 20× objective lens with Texas Red, FITC and DAPI filter sets.

### Flow cytometry analysis

Human iPSCs were dissociated using TrypLE solution (Thermo Fisher) then fixed and permeabilized for intracellular staining as described previously [5]. The fluorophore conjugated antibodies used in this protocol are listed in Table 2. Samples were analyzed by an AccuriC6 Flow Cytometry system (BD Biosciences).

### G-banded karyotyping

Karyotype analysis was performed by WiCell Research Institute (Madison, WI). Twenty randomly selected metaphases were selected and analyzed using G-banding method.

### Short tandem repeat (STR) analysis

STR analysis of the patient-derived fibroblasts and patient iPS cells was conducted by the Johns Hopkins University Genetic Resources Facility using a PowerPlex 18D Kit (Promega). The PCR products were electrophoresed on an ABI Prism^®^ 3730x1 Genetic Analyzer. GeneMapper^®^ v 4.0 software (Applied Biosystems) was used to analyze data.

### Mycoplasma detection

Mycoplasma status was assessed using the MycoAlert kit (Lonza) per manufacturer instructions. A ratiometric reading of < 0.9 indicates a mycoplasma negative sample.

### Testing for Sendai reprogramming vector clearance

Human fibroblasts (GM05659, Coriell) were transfected with Sendai virus for 4 days and used as the positive control. Total RNA was extracted using RNeasy Plus Mini Kit (Qiagen) and 1 μg of RNA was reverse transcribed into cDNA with Superscript^™^ III First-Strand Synthesis SuperMix kit. PCR was performed using Platinum II Hot-Start PCR Master Mix (ThermoFisher) and the amplifications were carried out as previously described (Li et al., 2019) using the primers listed in Table 2. Products were then loaded to the *E*-Gel^®^ 1.2% with SYBR Safe^™^ gel and imaged using a G: Box Chemi-XX6 gel doc system (Syngene).

### Teratoma formation assay

TRNDi008-A cells were dissociated from 6-well plates using 0.5 mM EDTA in DPBS. A suspension of 1 × 10^7^ cells in 400 μl medium supplied with 25 mM HEPES (pH 7.4) was kept on ice, and then added with a 50% volume (200 μl) of cold Matrigel (Corning, 354277). The mixture was injected subcutaneously into NSG mice (JAX No. 005557) at 150 μl per injection site. After 6–8 weeks, visible tumors were removed and fixed in 10% Neutral Buffer Formalin. The fixed tumors were embedded in paraffin and stained with hematoxylin and eosin.

## Supplementary Material

1

## Figures and Tables

**Table 1:** Characterization and validation.

Classification	Test	Result	Data
Morphology	Photography	Normal	Fig. 1 Panel A
Phenotype	Immunocytochemistry	SOX2, OCT4, NANOG, SSEA-4	Fig. 1 Panel A
	Flow cytometry	TRA-1-60 (99.97%); NANOG (93.26%); SSEA-4 (100%)	Fig. 1 Panel B
Genotype	Karyotype (G-banding) and resolution	46XY	Fig. 1 Panel C
		Resolution: 350–400	
Identity	Microsatellite PCR (mPCR) OR	Not performed	N/A
	STR analysis	18 sites tested, all sites matched	Available with the authors
Mutation analysis (IF APPLICABLE)	Sequencing	Hemizygous mutation of IDS	Fig. 1 Panel D
	Southern Blot OR WGS	N/A	N/A
Microbiology and virology	Mycoplasma	Mycoplasma testing by luminescence. Negative Teratoma with three germlayers formation. Ectoderm (neural tube); Mesoderm (smooth muscle); Endoderm (gut)	Supplementary Fig. S1
Differentiation potential	Teratoma formation		Fig. 1 Panel F
Donor screening (OPTIONAL)	HIV 1 + 2 Hepatitis B, Hepatitis C	N/A	N/A
Genotype additional info (OPTIONAL)	Blood group genotyping	N/A	N/A
	HLA tissue typing	N/A	N/A

**Table 2:** Reagents details

Antibodies used for immunocytochemistry/flow-cytometry			
	Antibody	Dilution	Company Cat # and RRID
Pluripotency Markers	Mouse anti-SOX2	1:50	R & D systems, Cat# MAB2018, RRID: AB_358009
Pluripotency Markers	Rabbit anti-NANOG	1:400	Cell Signaling Technology, Cat# 4903, RRID: AB_10559205
Pluripotency Markers	Rabbit anti-OCT4	1:400	Thermo Fisher, Cat# A13998, RRID: AB_2534182
Pluripotency Markers	Mouse anti-SSEA4	1:1000	Cell Signaling Technology, Cat# 4755, RRID: AB_1264259
Secondary Antibodies	Donkey anti-Mouse IgG (Alexa Fluor 488)	1:400	Thermo Fischer, Cat# A21202, RRID: AB_141607
Secondary Antibodies	Donkey anti-Rabbit IgG (Alexa Fluor 594)	1:400	Thermo Fischer, Cat# A21207, RRID: AB_141637
Flow Cytometry Antibodies	Anti-Tra-1-60-DyLight 488	1:50	Thermo Fischer, Cat# MA1–023-D488X, RRID: AB_2536700
Flow Cytometry Antibodies	Anti-Nanog-Alexa Fluor 488	1:50	Millipore, Cat# FCABS352A4, RRID: AB_10807973
Flow Cytometry Antibodies	anti-SSEA-4-Alexa Fluor 488	1:50	Thermo Fischer, Cat# 53-8843-41, RRID: AB_10597752
Flow Cytometry Antibodies	Mouse-IgM-DyLight 488	1:50	Thermo Fischer, Cat# MA1-194-D488, RRID: AB_2536969
Flow Cytometry Antibodies	Rabbit IgG-Alexa Fluor 488	1:50	Cell Signaling Technology, Cat# 4340S, RRID: AB_10694568
Flow Cytometry Antibodies	Mouse IgG3-FITC	1:50	Thermo Fischer, Cat# 11-4742-42, RRID: AB_2043894

Primers		
	Target	Forward/Reverse primer (5′-3′)
Sev specific primers (RT-PCR)	Sev/181 bp	GGA TCA CTA GGT GAT ATC GAG C/ACC AGA CAA GAG TTT AAG AGA TAT GTA TC
Sev specific primers (RT-PCR)	KOS/528 bp	ATG CAC CGC TAC GAC GTG AGC GC/ACC TTG ACA ATC CTG ATG TGG
Sev specific primers (RT-PCR)	Klf4/410 bp	TTC CTG CAT GCC AGA GGA GCC C/AAT GTA TCG AAG GTG CTC AA
Sev specific primers (RT-PCR)	C-Myc/523 bp	TAA CTG ACT AGC AGG CTT GTC G/TCC ACA TAC AGT CCT GGA TGA TGA TG
House-Keeping gene (RT-PCR)	GAPDH/197 bp	GGA GCG AGA TCC CTC CAA AAT/GGC TGT TGT CAT ACT TCT CAT GG
Targeted mutation analysis (PCR)	IDS (208insC)/357 bp	CGC ACA ATC TGT GCC ATC TG/TGG CTA GGC TGT TAA GGT GC
