# Supplementary figures and images for: Generation of an induced pluripotent stem cell line (TRNDi008-A) from a Hunter syndrome patient carrying a hemizygous 208insC mutation in the IDS gene
Source: Stem Cell Res. Author manuscript; Available in PMC 2019 Jul 20. (PMC6642610; doi:10.1016/j.scr.2019.101451)

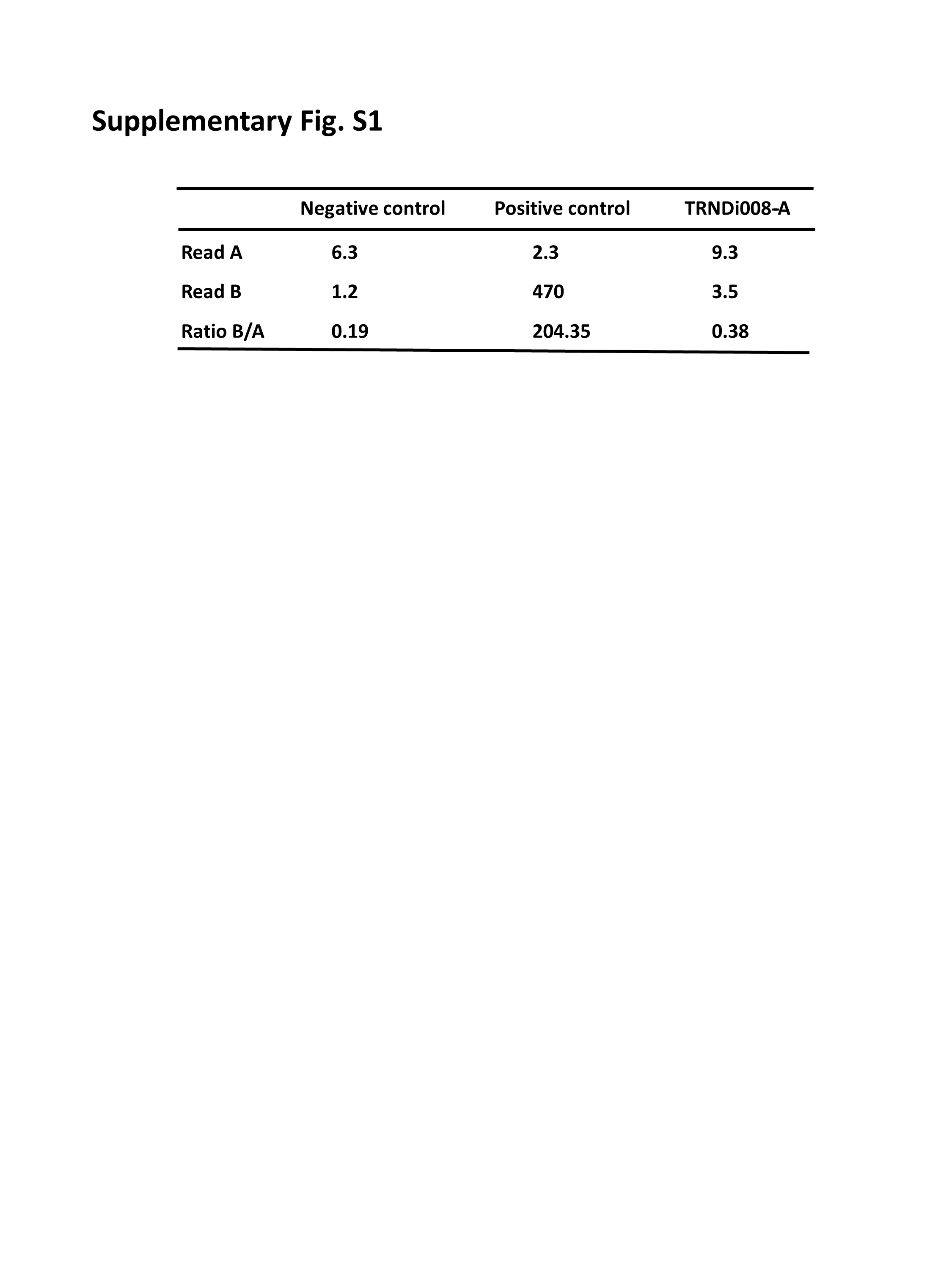

Supplement: 1 [file NIHMS1530899-supplement-1.tif]
